# Autophagy Is Impaired in Neutrophils from Streptozotocin-Induced Diabetic Rats

**DOI:** 10.3389/fimmu.2017.00024

**Published:** 2017-01-20

**Authors:** Wilson Mitsuo Tatagiba Kuwabara, Rui Curi, Tatiana Carolina Alba-Loureiro

**Affiliations:** ^1^Department of Physiology and Biophysics, Institute of Biomedical Sciences, University of São Paulo, São Paulo, Brazil

**Keywords:** neutrophil, diabetes type 1, autophagy, cell death and LC3B

## Abstract

We tested the hypothesis that changes reported on functions of neutrophils from streptozotocin-induced diabetic rats involve autophagy impairment. Wistar rats were rendered diabetic by streptozotocin injection (65 mg/kg, i.v.), and the measurements were carried out 2 weeks afterward. Neutrophils were collected through intraperitoneal cavity lavage after 4 h of i.p. oyster glycogen type 2 injection. Neutrophils cultured with PMA (20 nM) for 1 h were used for analysis of plasma membrane integrity, DNA fragmentation, and mitochondrial depolarization by flow cytometry; expression of Atg5, Atg14, Beclin1, LC3BII, and Rab9 by RT-PCR; the contents of caspase 3, LC3BII/LC3BI, and pS6 by western blotting; ATP content by fluorescence essay; reactive oxygen species production by chemiluminescence (Luminol), and autophagy by immunofluorescence tracking LC3B cleavage. Herein, neutrophils from diabetic rats had high DNA fragmentation, depolarization of mitochondrial membrane, low content of ATP, and high content of cleaved caspase 3 after PMA stimulation. Neutrophils from diabetic rats also had low expression of LC3B, failed to increase the expression of Rab9 and Atg14 induced by PMA stimulation. Neutrophils from diabetic animals also had low cleavage of LC3BI to LC3BII and do not present punctate structures that label autophagosomal membranes after stimulus. The changes of neutrophil function reported in diabetic rats do involve impaired autophagy. The suppression of autophagy in neutrophils from diabetic rats may be associated with the activation of the mTOR signaling as indicated by the high content of pS6.

## Introduction

Changes in neutrophil functions, such as chemotaxis (1), production of inflammatory mediators (2, 3), phagocytosis (4), bactericidal activity (5, 6), production of reactive oxygen species (ROS) (7, 8), and programed cell death (9), contribute to the high incidence of infections in diabetic patients. We, previously, reported that type I diabetes impairs neutrophil migration by decreasing the expression of adhesion proteins, e.g., intracellular adhesion molecule 1, production of prostaglandin E_2_ (PGE_2_), interleukin 1β, tumor necrosis factor α (TNF-α), cytokine-induced neutrophil chemoattractant 2α/β (CINC-2α/β), and interleukin 10 (3, 10–12).

During the apoptosis process, neutrophils exhibit marked morphological changes, such as cytoplasm condensation, organelle aggregations, nuclear chromatin cleavage, formation of apoptotic bodies, and reduction in cell volume (13, 14). Apoptotic neutrophils also have increased expression of pro-apoptotic B-cell lymphoma 2 (BCL-2) family members, activation of caspases, reduction of mitochondrial membrane potential, phosphatidylserine externalization on plasma membrane, and DNA fragmentation (15, 16). Autophagy plays an essential role in regulating cell death. It prevents apoptosis (17) by removing pro-apoptotic proteins, e.g., B-cell lymphoma-associated X and BCL-2 antagonist killer or by generating caspase inhibitors (18, 19). Autophagy is an evolutionary cellular degradation mechanism involved in cell homeostasis. It is mainly activated by nutrient starvation (20), oxidative stress (21), endoplasmic reticulum stress (22), and energy deficiency (23).

Macroautophagy is the major type of autophagy in which cytosol and organelles are sequestered within double-membrane vesicles (autophagosome) that deliver their contents to the lysosome/vacuole for enzymatic degradation and product recycling (24). Autophagosome formation is completed after the initiation, elongation, and expansion stages being controlled by autophagy-related genes (Atg) (25), Unc-51-like kinase, phosphatidylinositol 3-kinase (PI3K) and ATG12–ATG5–ATG16L1 complexes, and microtubule-associated protein light chain 3 (LC3) family members (26, 27). In mammalian cells, the conversion of LC3BI to LC3BII is a marker of autophagy (28).

Autophagy is regulated by changes in cell metabolism (29). The serine/threonine-protein kinase mammalian target of rapamycin (mTOR) is known to play a central role in cell survival, growth, proliferation, and cell metabolism by increasing various anabolic processes (e.g., lipids and proteins synthesis), and by inhibiting catabolic processes, such as autophagy (30, 31). This protein is activated whenever there is nutrient surplus in the cell, such as amino acids, free fatty acids (FFAs), and glucose (29, 30, 32). Activated mTOR phosphorylates the eukaryotic initiation factor 4E-binding protein 1 (4E-BP1) and the p70 ribosomal S6 kinase 1 (S6K1), which phosphorylates the ribosomal protein S6 and consequently regulates protein synthesis (33). Moreover, stimulation of mTOR leads to activation of sterol regulatory element-binding protein 1 (34) and of peroxisome proliferator-activated receptor-γ (PPARγ) (35) and so regulates lipid synthesis. mTOR activation may inhibi autophagy through Atg13, UNC-51-like kinase 1 (ULK1) phosphorylation. Phosphorylated Atg13 and ULK1 become inactive and then block the initiation of the autophagy process (29, 36–38).

Autophagy is involved in different steps of neutrophil defense against pathogens. Yano and Kurata (39) described that the mobilization of neutrophil lysosomes/endosomes and the release of their cargoes into the autophagic-vesicles contribute to the intracellular pathogen killing. LC3BII was detected in phagosomes from bone marrow-derived murine neutrophils (40). Mitroulis’ group reported that autophagy plays an important role in the phagocytosis-independent and phagocytosis-dependent microorganism killing in human neutrophils (41).

The information above led us to postulate that impaired autophagy may be involved in the changes of neutrophil functions in type-I diabetes. Autophagy was examined in neutrophils from streptozotocin-induced diabetic rats for 2 weeks. The following autophagy signs were measured: expression of LC3B, response of Atg5, Atg14, beclin-1, and Ras-related protein 9 (Rab9) to Phorbol 12-Myristate 13-Acetate (PMA), cleavage of LC3BI to LC3BII, content of pS6 and ATP levels. The participation of mTOR signaling was estimated by measuring its product, the pS6 content. Neutrophil function was assessed by measurement of apoptosis markers (DNA fragmentation, mitochondrial membrane depolarization, and caspase 3 activation) and PMA-stimulated ROS production.

## Materials and Methods

### Animals

Male Wistar rats weighing 200 ± 20 g (about 2 months of age) were obtained from the Department of Physiology and Biophysics, Institute of Biomedical Sciences, University of São Paulo. The rats were maintained at 23 ± 2°C under a cycle of 12 h light: 12 h darkness, being allowed free access to food and water. The Animal Ethical Committee of the Institute of Biomedical Sciences approved the experimental procedure of this study (number 148/2009).

### Induction of Type-1 Diabetes

The experimental type 1 diabetes state was induced by intravenous injection of streptozotocin (65 mg/kg b.w.) dissolved in citrate buffer, pH 4.2. Control rats were injected with citrate buffer. Forty-eight hours after streptozotocin injection, blood glucose levels above 200 mg/dL confirmed the diabetic state. Blood was taken from the tail and glucose measured by using a glucose meter (Roche Diagnostics Corporation, IN, USA). Alba-Loureiro et al. (3, 11, 12) used a similar protocol.

### Experimental Protocol of the Study

After 2 weeks of diabetes induction, fed rats were euthanized between 9:00 and10:00 a.m. Neutrophils were obtained 4 h after the intraperitoneal injection of 10 mL 1% (w/v) glycogen solution (oyster glycogen type II) (Sigma Chemical Co., St. Louis, MO, USA) in phosphate-buffered saline (PBS) by intraperitoneal lavage with 20 mL PBS. The cell suspension was centrifuged at 4°C (500 × *g* for 10 min). Rimele et al. (42) and Sturm et al. (43) used similar procedure.

### Cell Culture

Neutrophils were cultured for 1 h, with or without PMA (20 nM), in endotoxin-free RPMI 1640 medium containing 10% heat-inactivated fetal bovine serum (FBS) at 37°C in a 5% CO_2_ atmosphere.

### Analysis of Plasma Membrane Integrity

Neutrophils (1 × 10^6^) were centrifuged at 1,000 × *g* for 15 min at 4°C, and the pellet obtained was re-suspended in 500 µL PBS and incubated with propidium iodide (PI) (50 µg/ml) for 5 min. After incubation at room temperature, the cells were analyzed in a FACS Calibur flow cytometer (Becton Dickinson, San Juan, CA, USA) using the Cell Quest software (Becton Dickinson). The equipment was set to analyze live and dead cells, and the debris were identified but not quantified. Fluorescence was measured as described above using the FL2 channel (Orange-red fluorescence—585/42 nm). Martins de Lima et al. (44) and Levada-Pires et al. (45) used similar procedure.

### DNA Fragmentation Assay

Neutrophils (1 × 10^6^) were suspended in a solution containing 50 µg/mL propidium iodide, 0.1% sodium citrate, and 0.1% Triton X-100 that permeabilizes the cells and allows the incorporation of the dye in the DNA. The cells were then incubated for 30 min at room temperature. Fluorescence was measured and analyzed by flow cytometry as described by Nicoletti et al. (46).

### Mitochondrial Transmembrane Potential (ΔΨm) Measurement

Neutrophils (3 × 10^6^ cells/mL) were re-suspended in RPMI 1640 medium and incubated with 5,5′,6,6′-tetrachloro-1,1′,3,3′-tetraethyl-benzimidazolycarbocyanine iodide (JC-1) at 3.5 µM (Sigma Chemical Co, St. Louis, MO, USA) at 37°C for 15 min. A control experiment was performed by incubation with carbonyl cyanide 3-chlorophenylhydrazone (CCCP) at 10 nM (Sigma Chemical Co., St. Louis, MO, USA) for 15 min at 37°C. Cells were then washed and re-suspended in 500 µL PBS and analyzed immediately afterward. JC-1 fluorescence was assessed by flow cytometry using FL1 (Green fluorescence—530/30 nm) and FL2 (Orange-red fluorescence—585/42 nm) channels. Distributions of red and green fluorescences from JC-1 were displayed as two-color contour plot analysis. The data are expressed as the ratio of the mean fluorescence intensity.

### Luminol-Enhanced Chemiluminescence Assay of ROS Production

Luminol (500 µM) was added to neutrophil (1 × 10^6^ cells/mL) in Hank’s balanced salt solution supplemented with CaCl_2_⋅2H_2_O (1.26 M), MgSO_4_⋅7H_2_O (0.81 mM), and glucose (5.5 mM), at 37°C, in a final volume of 0.2 mL. Luminol (5-amino-2,3-dihydro-1,4-phthalazindione) is a chemical light amplifier. The chemiluminescence response was monitored in the presence or absence of PMA (160 nM) for 45 min, at 37°C, in a microplate luminometer (Synergy HT, Biotek, USA). NADPH oxidase complex inhibitor, diphenylene iodonium (DPI) at 10 µM and a PKC inhibitor, GF109203x (GFX) at 0.625 µM were used.

### Determination of ATP Content

Neutrophils (1 × 10^7^) were re-suspended in 100 µL trichloroacetic acid, and the intracellular ATP levels were determined using the ATP determination kit according to manufacturer’s instructions (Molecular Probes, Eugene, OR, USA).

### Western Blot Analysis

Cells (1 × 10^7^) were re-suspended in 60 µL Triton X100 lysis buffer containing protease and phosphatase inhibitors. Proteins were resolved in SDS-PAGE and transferred to nitrocellulose membranes. The membranes were blocked for 1 h at room temperature with 5% skim milk and incubated with the specific primary antibodies overnight. Following incubation with secondary antibody conjugated to horseradish peroxidase, the bands were identified using the enhanced chemiluminescence system (Amersham Biosciences). Ponceau staining was used as the inner control (47, 48). Immunoblots were scanned and quantified using the ImageJ^®^ software. Caspase 3, pS6, S6, and LC3BII polyclonal antibodies were purchased from Cell Signaling (Danvers, MA, USA).

### Immunofluorescence Essays

Neutrophils (2 × 10^4^) were left to adhere to slides with the aid of a FANEM centrifuge (Excelsa Flex 3400). Treatment with paraformaldehyde (at 4%), for 30 min, was used to fix the cells. Neutrophils were permeabilized with 3% BSA–0.05% saponin solution for 30 min. After permeabilization, cells were blocked with 10% FBS for 1 h. Cells were incubated with LC3BI/II primary antibody (1:200) (Cell Signaling, Danvers, MA, USA) in a humidified chamber overnight. After overnight labeling with the primary antibody, neutrophils were incubated with fluorescent secondary antibody (1:10,000) (Donkey anti-Rabbit Alexa Fluor 488, Jackson ImmunoResearch, West Grove, PA, USA) for 1 h. Slides were then mounted with DAPI (4′,6- diamidino-2-phenylindole) ProLong^®^ Gold Mountant (Molecular Probes, Thermo Fisher Scientific, USA). Slides were evaluated in a multiphoton microscope LSM 780 NLO (Zeiss, Oberkochen, Germany). Analysis was performed in FIJI Image J software, cell by cell (at least 30 cells per condition per animal), and results represent the mean of three independent experiments. The control group Mean Intensity of Fluorescence (MIF) normalized the MIF of studied groups.

### Real-time Polymerase Chain Reaction (RT-PCR)

Total RNA was obtained from 1 × 10^7^ neutrophils by the guanidine isothiocyanate extraction method using TRIzol^®^ Reagent (Invitrogen, Carlsbad, CA, USA) (49) followed by isolation using RNeasy mini kit (Qiagen). The purity was assessed by the 260/280 nm ratio and the quantity measured at 260 nm. The cDNA was synthesized from total RNA (1.0 µg) using the High Capacity kit (Invitrogen). The following primers were used: Atg14 (5′-TGCCGAACAATGGGGACTAC-3′ and 5′-AGGCAGGGTTGTTATGCTCC-3′), Atg5 (5′-CTCAGCTCTGCCTTGGAACA-3′ and 5′- GTGAGCCTCAACTGCATCCT-3′), Beclin (5′-AGCACGCCATGTATAGCAAAGA-3′ and 5′-GGAAGAGGGAAAGGACAGCAT-3′), LC3II (5′-CCAAGCCTTCTTCCTCCTGG-3′ and 5′- TCTCCTGGGAGGCATAGACC-3′), and Rab9 (5′-CCAATGTTGCTGCTGCCTTT-3′ and 5′-GAGTTTGGCTTGGGCTTTCG-3′). Real-time PCR analysis was performed using the SyBR Green JumpStart kit (Sigma Aldrich) in a Rotor Gene 6000 equipment (Corbett Research, Mortlake, Australia). Gene expression was performed by 2^−ΔΔCT^ using RPL37a gene (5′-CGCTAAGTACACTTGCTCCTTCTG-3′and 5′-GCCACTGTTTTCATGCAGGAAC-3′) as inner control.

### Data Analysis

Results are presented as mean ± SEM. Statistical significance was assessed by two-way ANOVA followed by the Bonferroni post-test. *p* ≤ 0.05 was considered statistically significant.

## Results

As compared to controls, diabetic rats exhibited a significant reduction in body weight gain during the experimental period as expected, 67 ± 4 g for the control vs 12 ± 6 g for the diabetic groups (*p* < 0.0001); and the blood glucose levels were significantly elevated: 113 ± 3 mg/dL for the control and 457 ± 21 mg/dL for the diabetic groups (*p* < 0.0001). Neutrophil counts were performed 4 h after intraperitoneal injection of 10 mL (1%, w/v) oyster glycogen type II solution. The number of cells that migrated to the peritoneal cavity (×10^7^; mean ± SEM) was: 12.3 ± 2.7 for the control group and 6.7 ± 1.8 (*p* < 0.0001) for the diabetic group.

**Figure 1:**
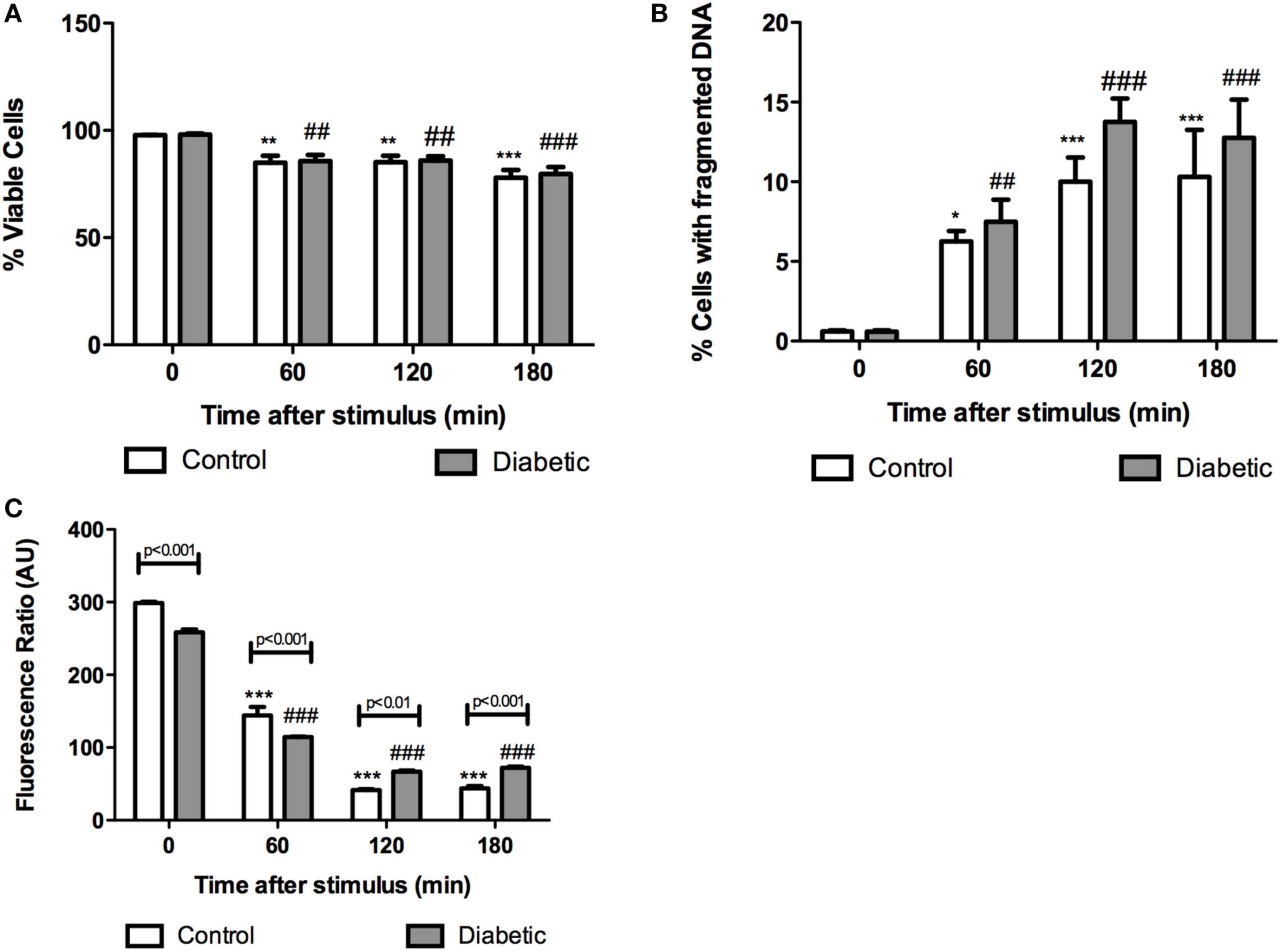
**Cell integrity, DNA fragmentation, and mitochondrial transmembrane potential in neutrophils from diabetic and control rats at different time points after PMA stimulus**. No differences in cell integrity and DNA fragmentation were observed between groups at the different time points. Neutrophils from the diabetic group had depolarization of mitochondrial transmembrane potential before and after 60 min PMA stimulus. Graphs **(A)** and **(B)** present the percentage of gated cells. Graph **(C)** represents the red/green fluorescence ratio of JC1. Analyses were performed in a FACSCalibur flow cytometer. Results are presented as mean ± SEM of six animals per group. (*) indicates *p* < 0.05 vs the control group before PMA stimulus; (**) indicates *p* < 0.01 vs the control group before PMA stimulus; (***) indicates *p* < 0.001 vs the control group before PMA stimulus. (##) indicates *p* < 0.01 vs the diabetic group before PMA stimulus; (###) indicates *p* < 0.001 vs the diabetic group before PMA stimulus.

**Figure 2:**
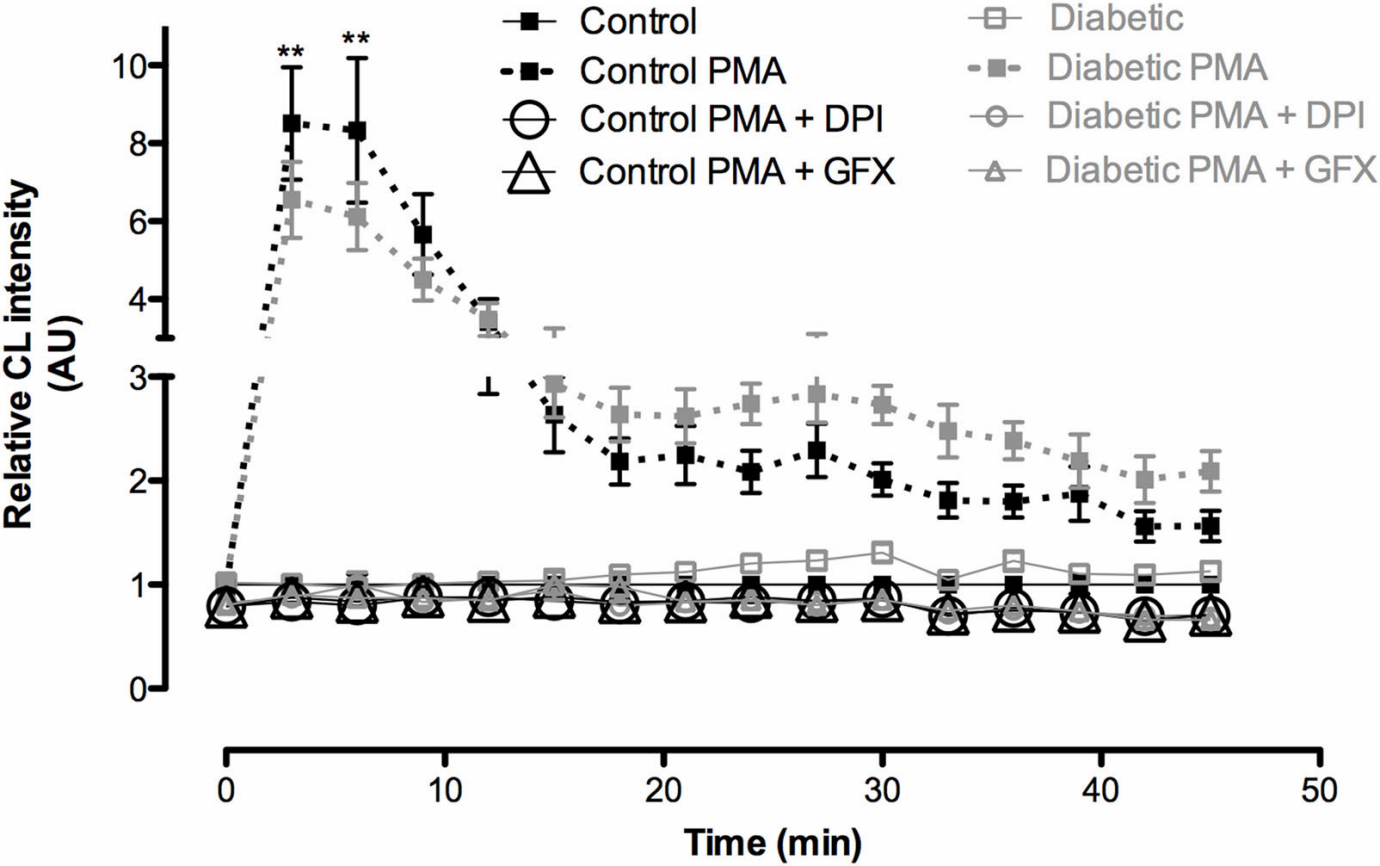
**Reactive oxygen species (ROS) production by neutrophils after PMA stimulus**. Neutrophils from diabetic rats produced less ROS than the ones from controls. No difference was observed between groups without PMA stimulus. GFX and DPI abolished the production of ROS. Results represent relative chemiluminescence intensity ± SEM from 12 animals per group. (**) indicates *p* < 0.01.

We, first, investigated the time course feature of neutrophil death after PMA (20 nM) treatment. The 60-minute period was then established as the time point in which the cell was not yet dead but the cell death process had possibly already been triggered. In this time point, neutrophils from both groups had decreased plasma membrane integrity (Figure 1A), increased DNA fragmentation (Figure 1B), and mitochondrial membrane depolarization (Figure 1C). Neutrophils from the diabetic group had significant mitochondrial membrane depolarization even in the absence of PMA (basal) and higher degree of depolarization after 60-min PMA stimulus as compared to the control group (Figure 1C). PMA is a strong pharmacological agent that acts *via* PKC to promote NADPH oxidase complex assembly and oxidative burst. Reduced ROS production in neutrophils from STZ diabetic rats was observed (Figure 2). GFX and DPI, inhibitors of NADPH oxidase, fully inhibited ROS production in neutrophils from both groups, indicating that this enzyme complex is the main site of ROS production during PMA stimulation.

**Figure 3:**
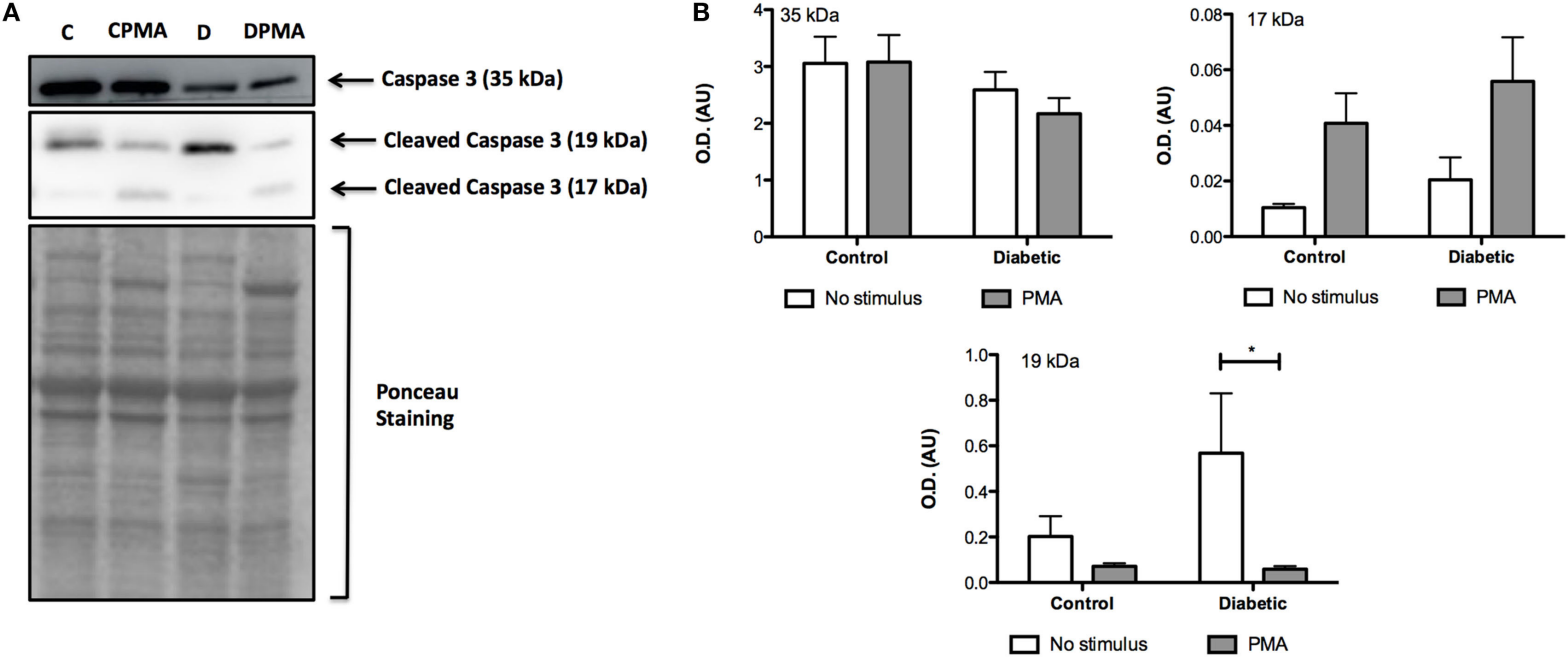
**Content of non-cleaved (35 kDa) and cleaved (17 and 19 kDa) caspase 3**. PMA treatment augmented the content of cleaved caspase 3. **(A)** No differences were observed in the non-cleaved caspase 3. No statistical difference was observed between groups. **(B)** Neutrophils from diabetic rats without stimulus had higher content of 19 kDa cleaved caspase 3. **(A,B)** Graphs represent mean OD ± SEM of the bands from six animals per group. (*) indicates *p* < 0.05.

**Figure 4:**
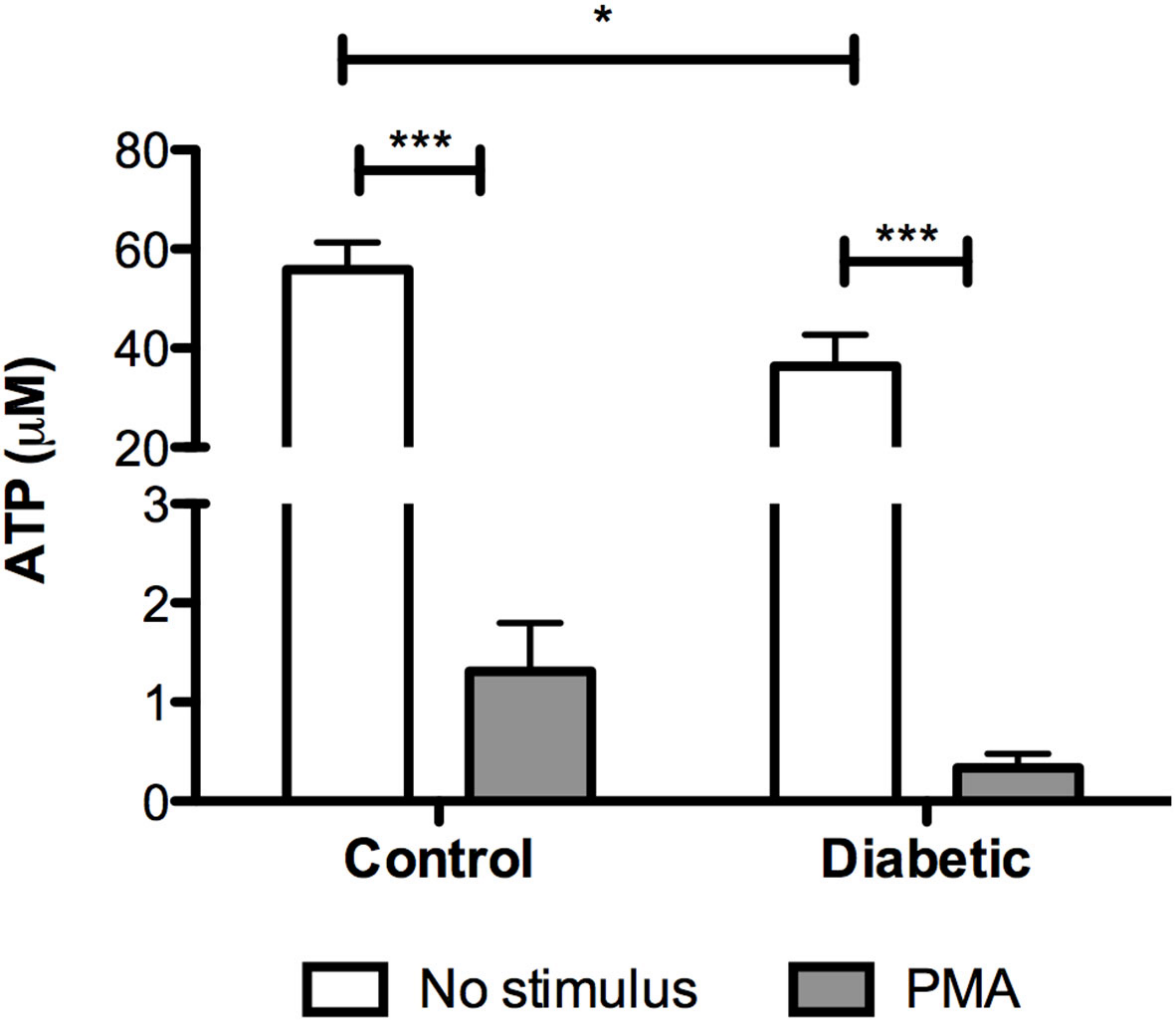
**ATP content in neutrophils before and after PMA stimulus**. Neutrophils from diabetic had lower content of ATP. PMA decreased the content of ATP in both groups. Results presented as concentration of ATP (μM) ± SEM from four animals per group. (*) indicates *p* < 0.05; (***) indicates *p* < 0.001.

Mitochondria depolarization has been associated with mitochondrial membrane pore opening and release of Cytochrome *c*, which activates caspase-3 in the cytosol. Caspase-3 cleavage (activation) was increased in neutrophils from diabetic rats (Figure 3). Intracellular ATP content was measured before and after PMA stimulation (Figure 4). Neutrophils from diabetic rats had 35% lower ATP content before PMA stimulus (μM, as mean ± SEM of four animals; 55.8 ± 5.42 for control, and 36.4 ± 6.31 for diabetic) and 74% decrease in ATP content after PMA stimulus (μM, as mean ± SEM of four animals; 1.30 ± 0.49 for control, and 0.34 ± 0.14 for diabetic) when compared to cells of the control group.

**Figure 5:**
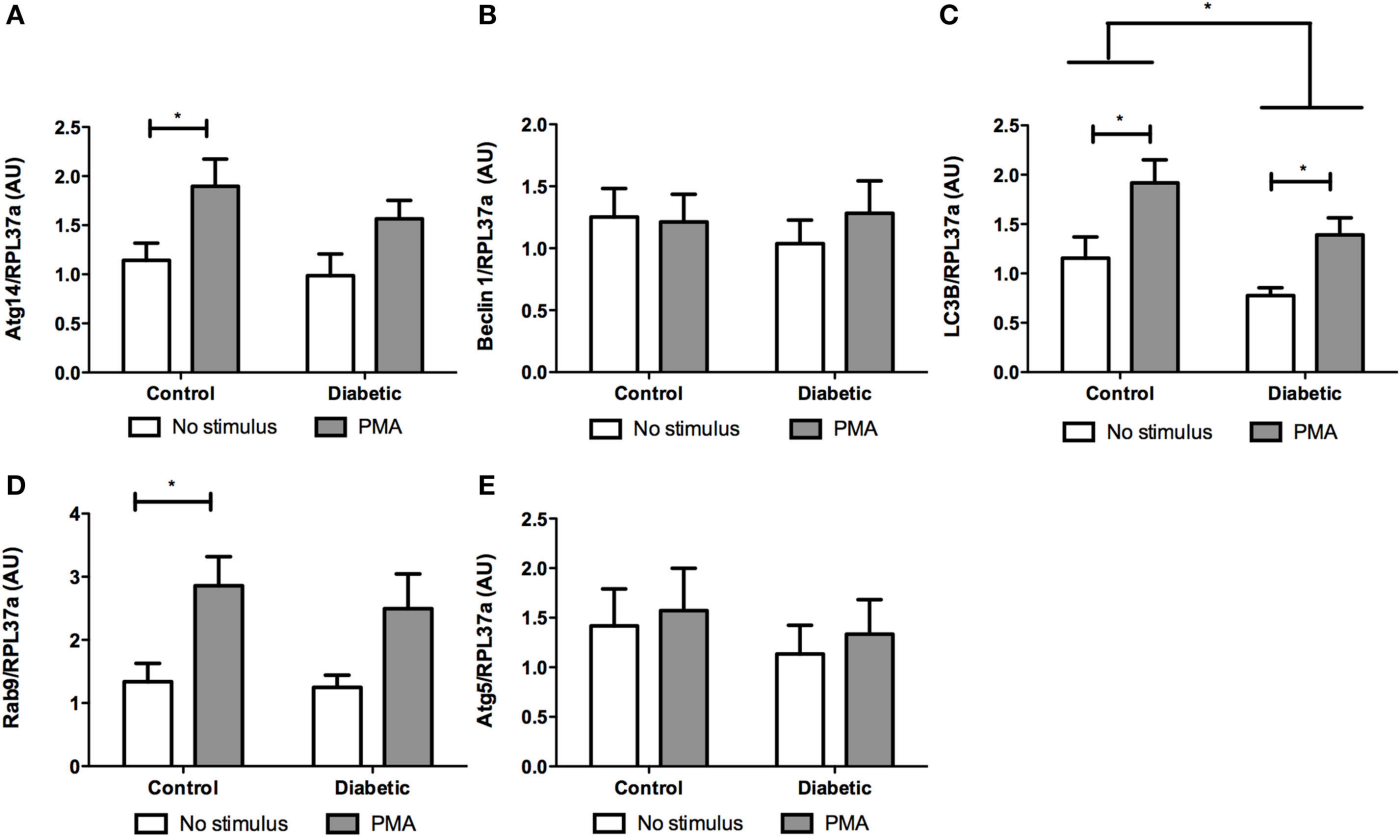
**Effect of reactive oxygen species on expression of genes related to autophagy**. PMA augmented the expression of Atg14 **(A)** and Rab9 **(D)** in the control group and no statistic difference was observed in neutrophils from diabetic rats. LC3B expression was lower in the diabetic group **(C)**. There were no differences in Beclin 1 **(B)** and Atg5 **(E)** expression. Results represent the relative expression of the genes ± SEM from 12 animals per group. RPL37a was used as a reference control gene. (*) indicates *p* < 0.05.

**Figure 6:**
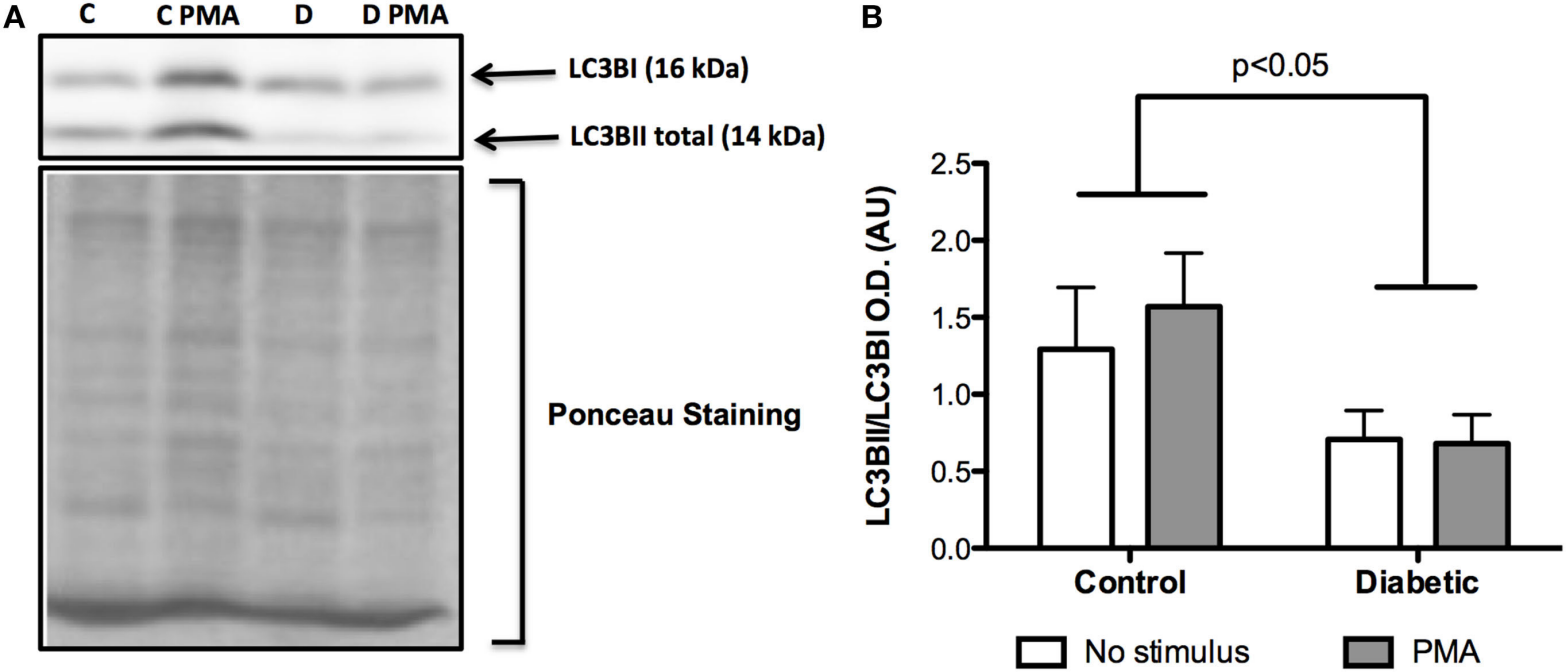
**Contents of LC3BI and LC3BII in neutrophils before and after PMA stimulus**. Neutrophils from diabetic rats had low cleavage of LC3BI to LC3BII when compared to the control group **(A)**. Graph **(B)** presents mean OD ± SEM of the bands from six animals per group.

**Figure 7:**
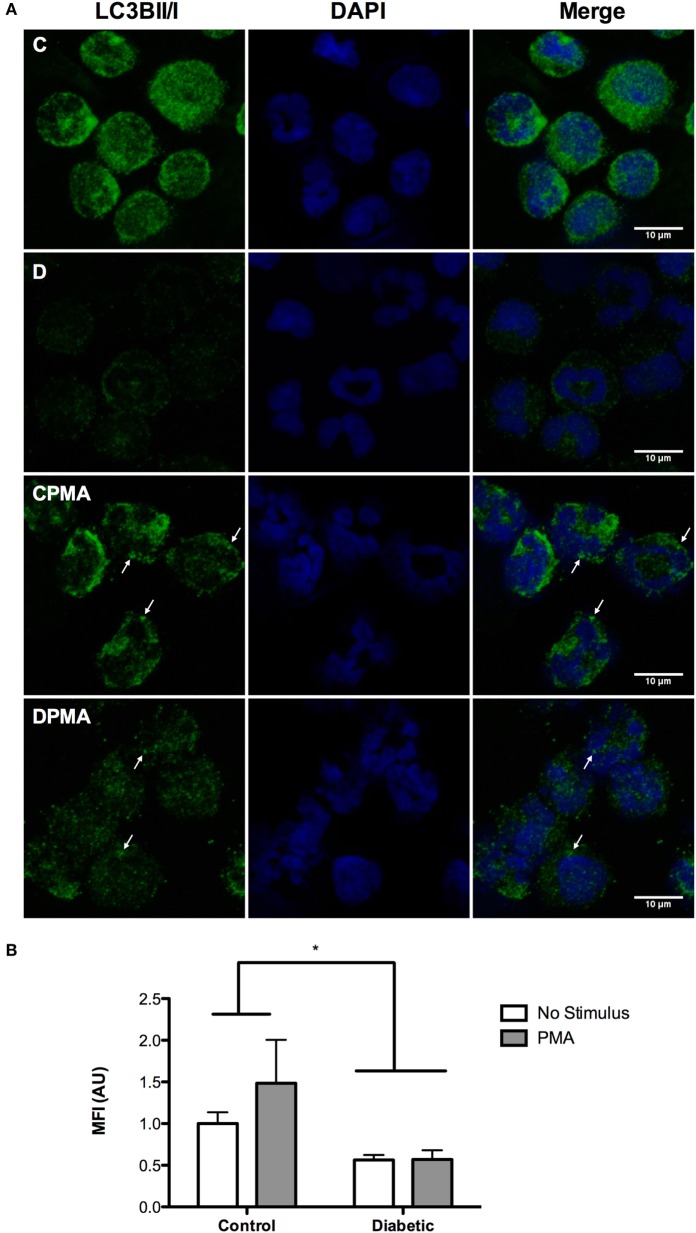
**Immunofluorescence of LC3BII/I in neutrophils before and after PMA stimulus**. Neutrophils from diabetic rats had low content of LC3BII/I before and after PMA stimulus when compared to the control group **(A)**. Graph **(B)** presents MIF ± SEM of at least 30 cells per group. Analysis was performed cell by cell. The values were normalized by the control group. Green: LC3BII/I; blue (nucleus): DAPI. (*) indicates *p* < 0.05.

**Figure 8:**
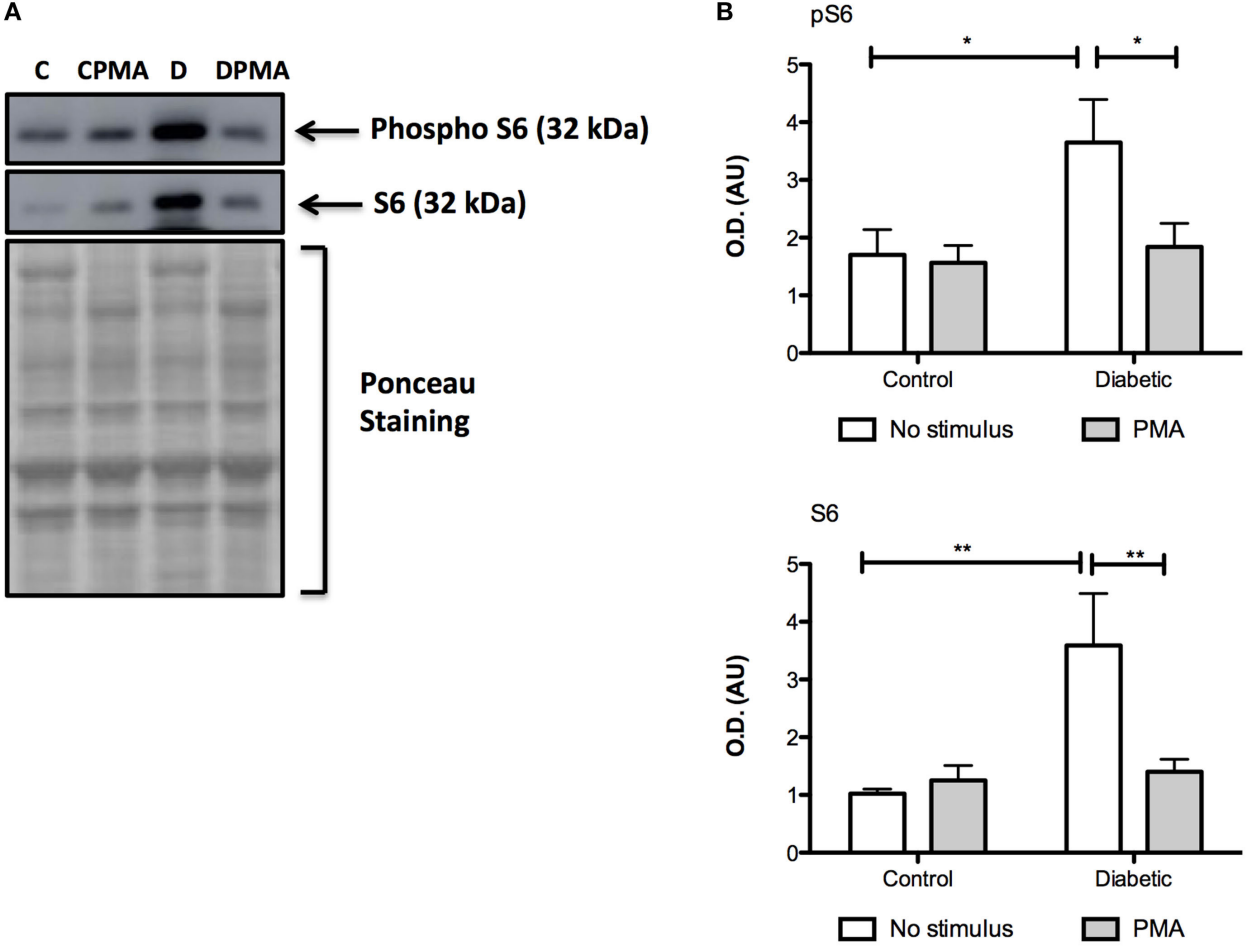
**Contents of pS6 and S6 in neutrophils before and after PMA stimulus**. Neutrophils from diabetic rats had high contents of pS6 and S6 when compared to the control group **(A)**. Graphs **(B)** present mean OD ± SEM of the bands from six animals per group. (*) indicates *p* < 0.05; (*) indicates *p* < 0.01.

Neutrophils from diabetic rats had lower expression of LC3B before and after PMA stimulus when compared to the control group (Figure 5C). The expression of Rab9 (Figure 5D) and Atg14 (Figure 5A) was increased in response to PMA in the control group but did not have any difference in neutrophils from diabetic rats as compared with non-stimulated cells. The expressions of Atg5 (Figure 5E) and Beclin1 (Figure 5B) remained unchanged in the experimental conditions herein reported. Autophagy involves cytosolic LC3BI proteolytic cleavage and lipidation to form the membrane-bound LC3BII. The content of LC3BII (an autophagy marker) was measured by immunoblot analysis. The content of LC3BII was lowered in neutrophils from diabetic rats (Figure 6). The activation of autophagy was also investigated by the intracellular localization of LC3B using immunofluorescence. This protein is redistributed from a diffuse cytoplasmic pattern to form punctate structures that label pre-autophagosomal and autophagosomal membranes when autophagy is triggered (50). Neutrophils from diabetic rats with or without stimulus did not present punctate staining whereas it can be seen in PMA-stimulated neutrophils from control rats (Figure 7). Neutrophils from diabetic rats had higher content of phosphorylated S6 (pS6) when compared with neutrophils from controls (Figure 8), evidencing mTOR activation.

## Discussion

Evidence is presented, herein, that autophagy is impaired in neutrophils from diabetic rats. Malfunctioning mitochondria and decreased ATP content have been associated with impairment in autophagy and shortened neutrophil life span as indicated by the increased content of cleaved caspase 3. This feature of neutrophils from diabetic rats is probably a consequence of the apoptotic process activation through autophagy suppression (51).

NADPH oxidase-derived ROS are involved in the phagocytosis and autophagy pathways during the recruitment of LC3B to phagosomes in phagocytic cells (52, 53). PMA has been reported to induce autophagy in neutrophils due to increased oxidative burst *via* NADPH oxidase (41). We described, herein, that neutrophils from diabetic rats produce less ROS that leads to a decrease in microbial killing capacity, activation of ion channels, and protein modifications (54, 55). In fact, autophagy-deficient mice have reduced inflammatory potential of neutrophils as consequence of reduced myeloperoxidase (MPO) activity and ROS production through NADPH oxidase (56).

Low levels of ATP in neutrophils from diabetic rats might be a consequence of the respiratory chain loss-of-function induced by mitochondrial membrane depolarization. Mitochondrial dysfunction in PBMC and endothelial cells has been described in diabetic states (57–60). Alba-Loureiro et al. reported decreased glutamine consumption, unchanged glucose uptake, and augmented oxidation of FFAs in neutrophils from STZ-induced diabetic rats (11). FFAs easily cross plasma membrane and are oxidized in the mitochondria. However, FFA overload, particularly saturated FFA, promotes mitochondrial uncoupling and inhibition of the respiratory chain leading to organelle dysfunction (61). The increase of FFA oxidation in neutrophils from diabetic rats might then be associated with mitochondrial dysfunction (depolarization) and so decreased intracellular ATP content. Low levels of ATP increase the DNA fragmentation susceptibility and so cell death through increased oxidative stress (62). FFAs also increase inner mitochondrial membrane permeability with consequent release of pro-apoptotic proteins to cytosol (61) that then prompt apoptosis in neutrophils from diabetic rats.

Autophagy is inhibited by mTOR during the condition of nutrient surplus (63). Neutrophils from diabetic rats are constantly exposed to an overload of nutrients that exist in the plasma of the diabetic individuals, mainly composed by glucose and FFA (64–66). FFAs cross the plasma membrane freely due to their hydrophobicity and activate mTOR as observed in other cell types (32). Glucose also activates mTOR (29, 32). However, glucose has limited entry into leukocytes since these cells present the glucose transporter 1 (GLUT1). GLUT1 has low *K*_m_ (1–2 mM), and so, once the transport is saturated, there is no further glucose entry into the cells despite its levels increase in plasma (67).

Reactive oxygen species are known to inhibit mTOR in different cell types (68, 69). After PMA stimulus, a decrease of pS6 levels was observed in neutrophils from the diabetic group. This evident decrease was not observed in neutrophils from the control group, mainly because, differently from diabetic rats, there is no overload of nutrients in plasma of fasted healthy animals.

Neutrophils from diabetic rats fail to trigger autophagy as indicated by low expression of the LC3B and reduced cleavage of LC3BI to LC3BII. Bhattacharya et al. (70) described an important role of autophagy for neutrophil functions in inflammation and autoimmune diseases. Bone marrow-derived neutrophils from Atg7 and Atg5 knockout mice have reduced degranulation of primary, secondary, and tertiary granules, mainly due to decreased neutrophil granule fusion to phagosomes (56). Macrophages from Atg5 knockout mice have increased susceptibility to death indicating that autophagy reduces advanced atherosclerosis induction of macrophage death (71).

In conclusion, we can speculate that changes observed in neutrophils (e.g., increased apoptosis and ROS production) from diabetic rats could be associated with suppressed autophagy by mTOR over activation leading to cell death.

## Author Contributions

WK: experiments design and development and article writing; RC: article review; TA-L: experiments design and development, article writing, and article review.

## Conflict of Interest Statement

The authors declare that the research was conducted in the absence of any commercial or financial relationships that could be construed as a potential conflict of interest.
