# Supplementary figures and images for: Autophagy Is Impaired in Neutrophils from Streptozotocin-Induced Diabetic Rats
Source: Front Immunol. 2017 Jan 20;8:24. doi: 10.3389/fimmu.2017.00024 (PMC5247474; doi:10.3389/fimmu.2017.00024)

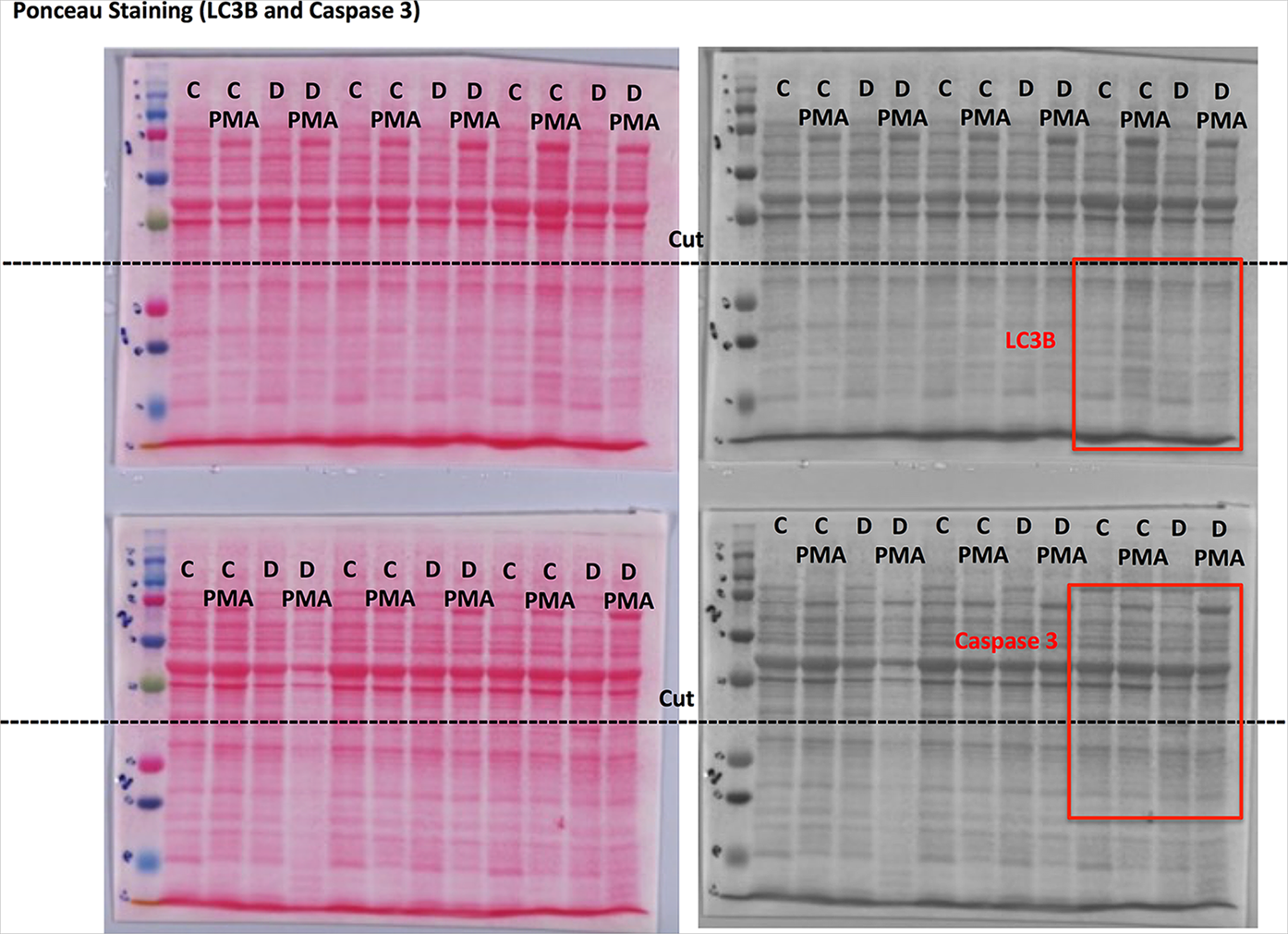

Supplement: Supplementary file 1 [file image_1.tiff]

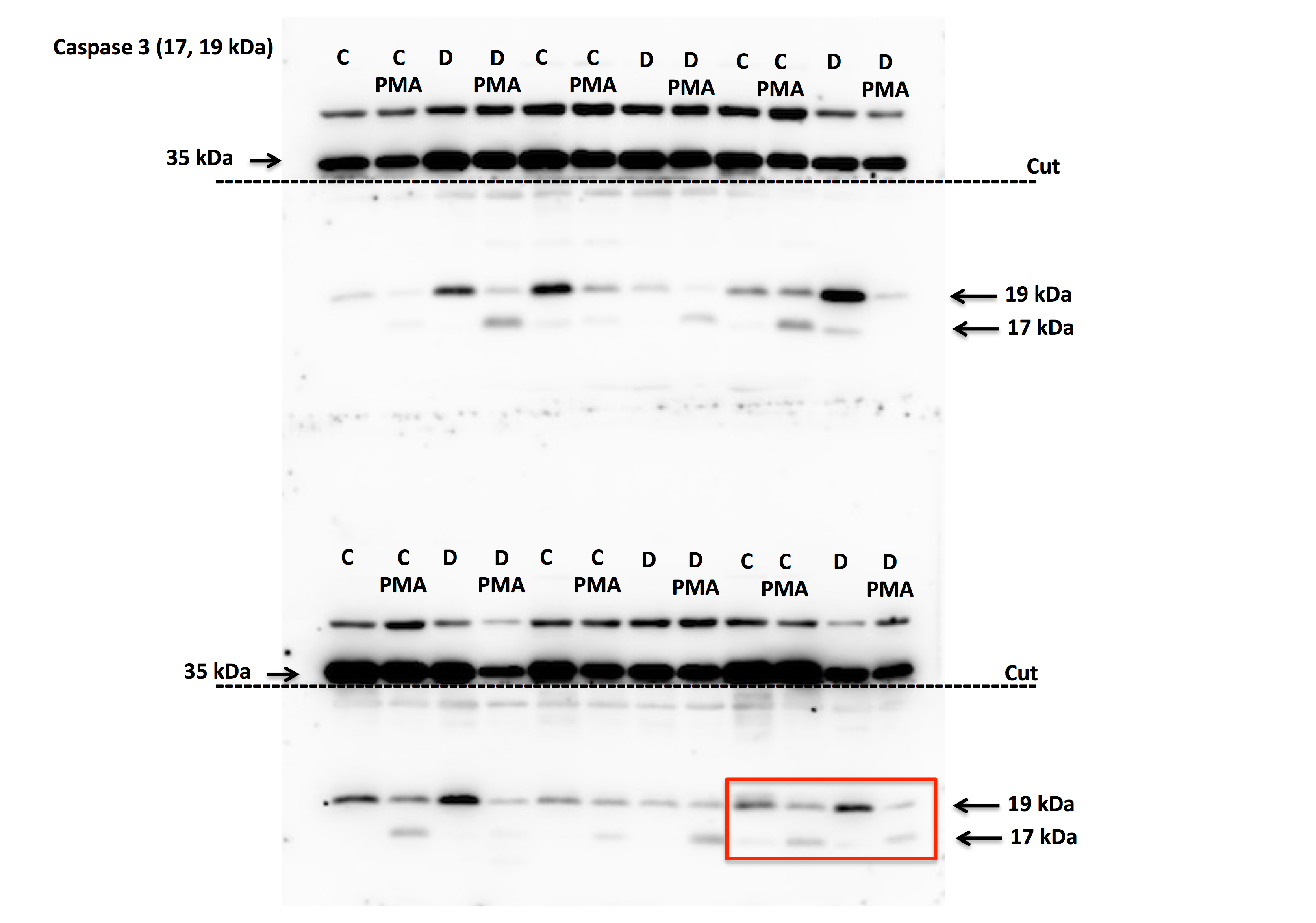

Supplement: Supplementary file 2 [file image_2.tiff]

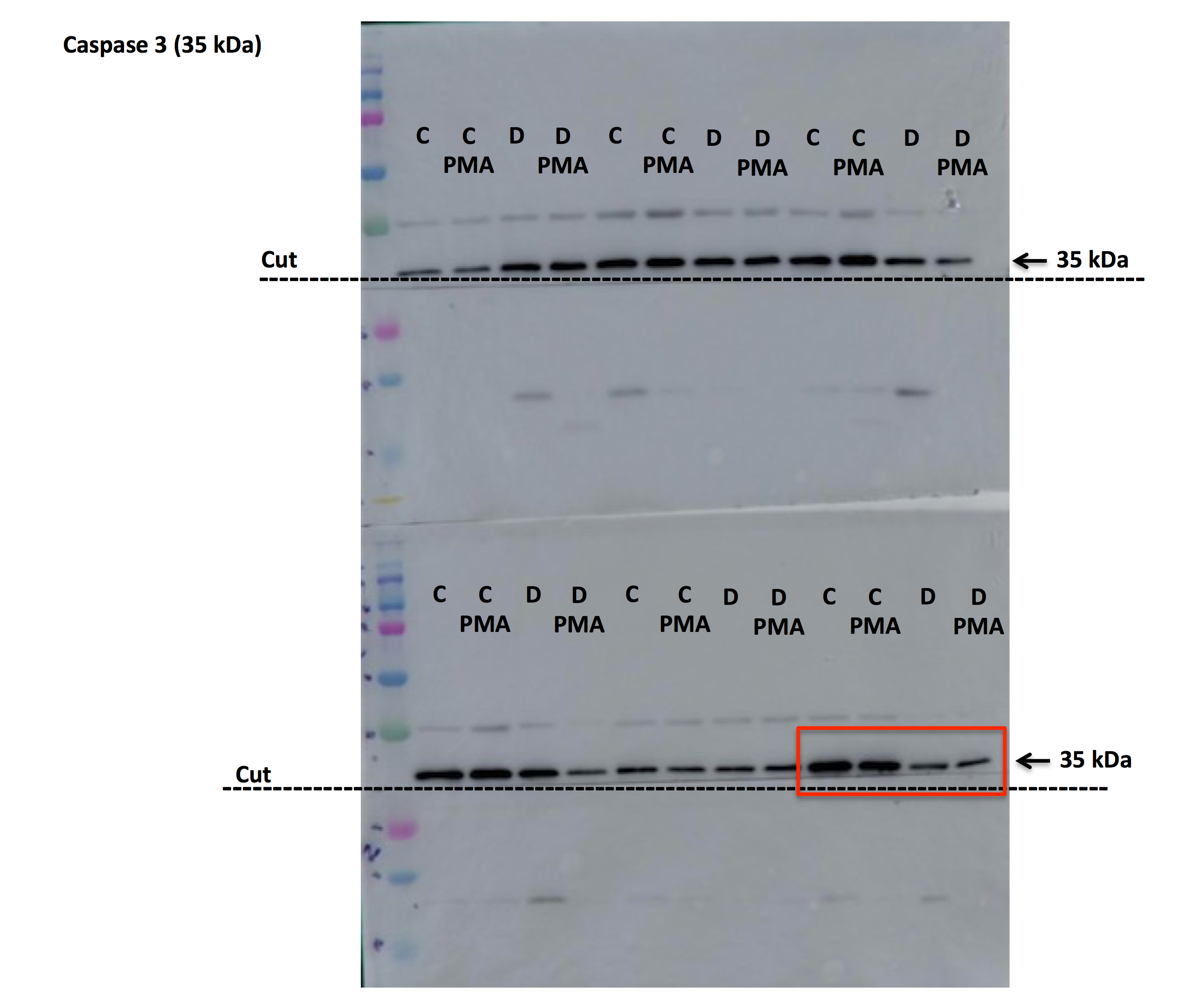

Supplement: Supplementary file 3 [file image_3.tiff]

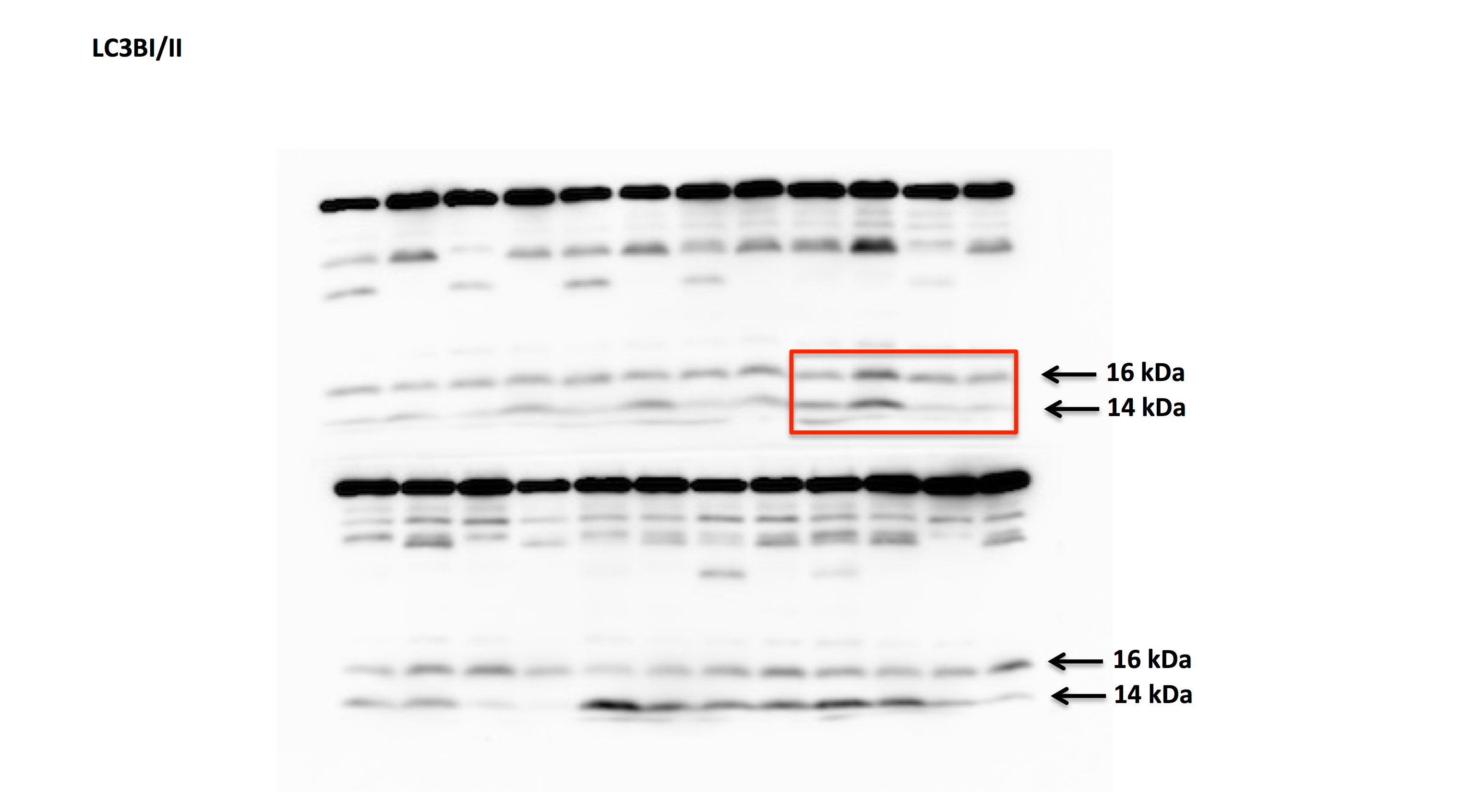

Supplement: Supplementary file 4 [file image_4.tiff]

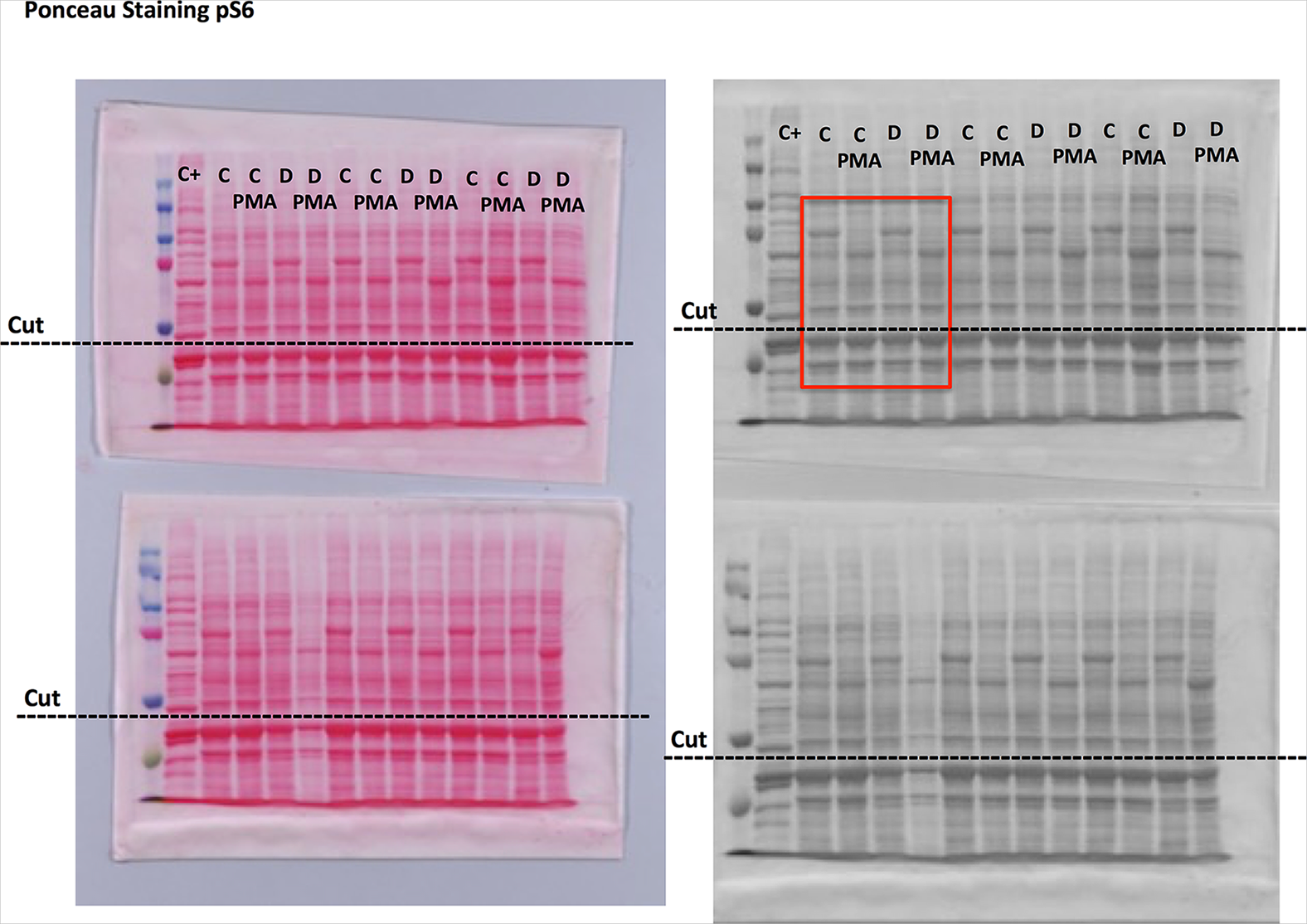

Supplement: Supplementary file 5 [file image_5.tiff]

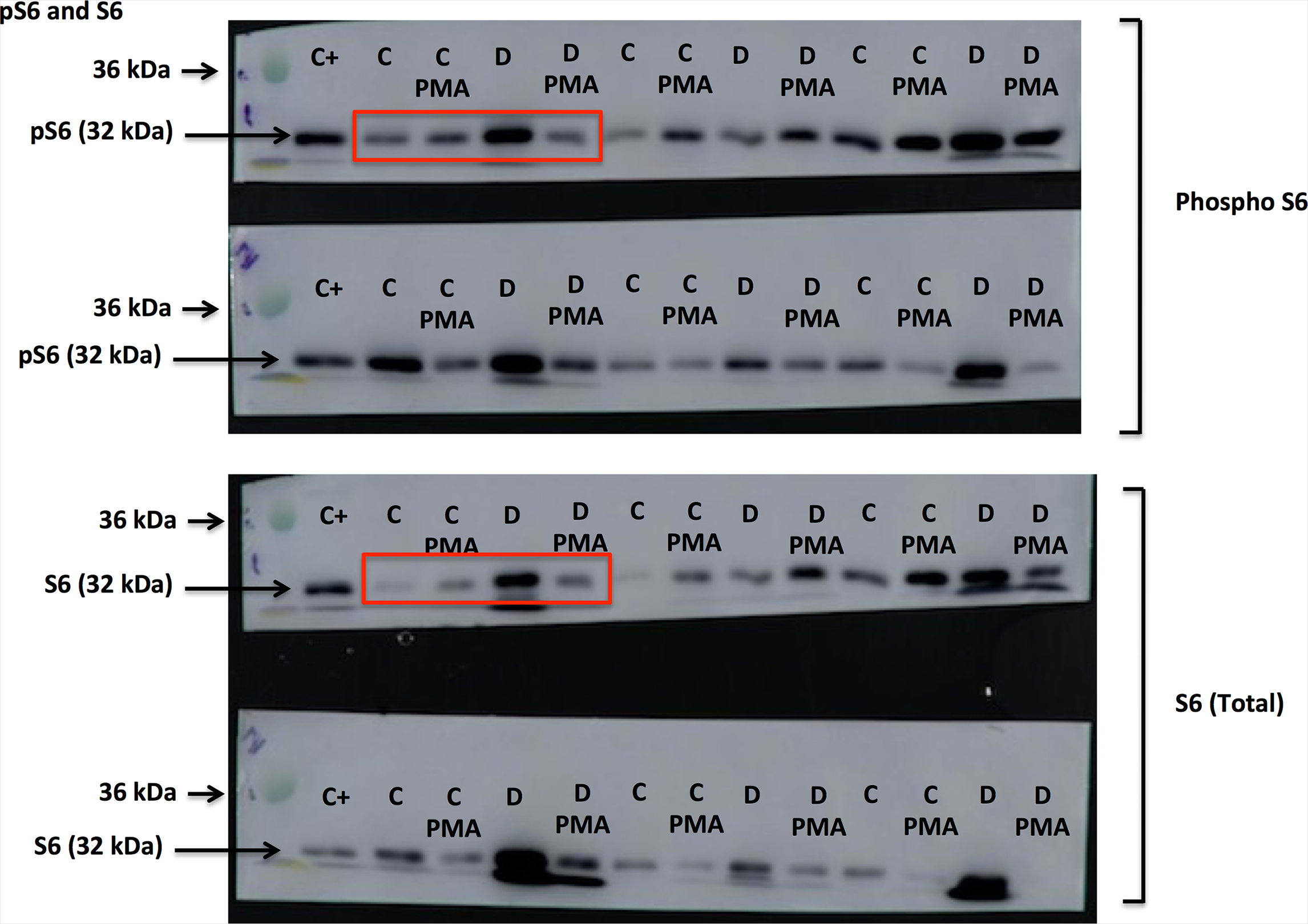

Supplement: Supplementary file 6 [file image_6.tiff]
